# A Multidimensional Assessment Model Using RE–3DSG Sensors on Net ES and GVR for Sustainable and Smart Cities

**DOI:** 10.3390/s20051259

**Published:** 2020-02-25

**Authors:** Jia Jia, Xuefei Wu

**Affiliations:** College of Horticulture & Forestry Sciences of Huazhong Agricultural University, Wuhan 430070, China; jiajia08147@163.com

**Keywords:** RE–3DSG sensors, Net ES, GVR, sustainable and smart cities

## Abstract

With the rapid accumulation of population and industry, the urban service efficiency requirements in building sustainable and smart cities are increasingly becoming higher. However, current environmental assessment methods require large amounts of data, long assessment cycles, and tedious assessment processes; thus, they cannot quickly respond to the rapidly changing urban green space. To resolve the above problems, we present a multidimensional model for sustainable and smart cities equipped with RE–3DSG sensors to detect the real experience of residents and the three–dimensional structure of the urban green space. RE–3DSG sensors consist of two parts: The net ecosystem service (Net ES) and green volume ratio (GVR), where Net ES provides a solution consisting of runoff control, air purification, cooling, carbon sequestration, noise reduction, and recreational area establishment, while GVR assesses the spatial structure of urban built environment plant clusters. By implementing the proposed model, it is proven that it can assist users (usually decision makers in government departments) to improve the decision–making efficiency and increase the satisfaction of residents with urban green spaces, thereby achieving the goal of building a sustainable and smart city.

## 1. Introduction

With urbanization and population growth, it is becoming increasingly necessary to formulate sustainable growth plans for cities. Many cities worldwide are actively seeking smart growth methods to solve environmental problems in dense cities, thereby improving the efficiency of urban services [1,2,3]. Smart cities based on IoT technology are an inevitable trend aimed at increasing environmental sustainability in urban development while enhancing economic prosperity and social equity. The world is gradually urbanizing, small cities are developing rapidly, large cities are becoming increasingly larger, and the population is increasing much faster. According to the forecast of the United Nations for 2050, the proportion of people living in cities is expected to reach 66.4%, an increase of 22% over the present. While more than half of the urbanization process has provided more convenient infrastructure to residents, it has also compressed the green infrastructure (GI) space, causing its size and variety to decrease, and the efficiency of urban services continues to decline [4]. Therefore, it is urgent to develop an effective means to improve and optimize urban green spaces. However, due to the differences in population, development status, and geographical location of cities, a smart growth plan tailored to each city must be introduced. The smart urban growth plan can be used to solve the problems of the increasing population and development of the city plans for the next 30 years.

Currently, environmental assessment methods for sustainable and smart cities can be roughly divided into four types: Multifactor overlay methods for urban green space assessment [5,6], urban green space assessment based on the spatial graph theory (MSPA) [7], urban green space assessment combined with land use (LUCC) [8], urban green space assessment based on traditional ecological patch–corridor–matrix models [9], and pressure–state–response models [10]. The above traditional methods have three shortcomings. First, they only focus on the total amount of the urban environment basing on objective facts, rather than basing it on the feelings of the real, urban residents or the structure type of urban green space. Second, their evaluation processes often require land use data, urban climate data, and vegetation type data, while those require huge amounts of data and are difficult to obtain. Third, the evaluation cycle is long, such as “Survey and Assessment of ten–year Change of National Ecological Environment (2000–2010)“ could last 10 years, and the implementation is difficult. In view of the problems existing in the previous research, the urban green space assessment method proposed by our work has gone one step further with the following characteristics: First, we are focusing on the specific quality and effect of the urban environment, and the assessment is based on the dynamic monitoring of the ecological environment and the real feelings of urban residents. Second, the data required for the process come from public government documents, and the required data are authoritative and easier to obtain. Last, the evaluation cycle can be flexibly changed with the evaluation needs and is easy to implement. Therefore, our work outperforms the traditional method on responding speed and can dynamically act quickly according to the rapid changing urban green space.

To construct the sustainable and smart cities, various IoT–based technologies e.g., facilities management [11], architecture [12,13], interior design [14], energy and environmental design [15], have been explored in related fields. Yet, one major shortcoming of the stand–alone version of the simulation evaluation software is that when cities are under operation, the results may be affected by unexpected factors, including uncertain and complex urban green space and personnel movements. To overcome the above limitation, this research proposes a multidimensional assessment model, which uses RE–3DSG sensors to detect the real resident experience and the three–dimensional structure of the urban green space. The net ecosystem service (Net ES) and green volume ratio (GVR) are two major parts of the model, where Net ES provides a solution consisting of runoff control, air purification, cooling, carbon sequestration, noise reduction, and recreational area establishment, while GVR assesses the spatial structure of urban built environment plant clusters. The model not only retains the advantages of the simulation evaluation software based on IoT technology to improve work efficiency and save manpower and material resources, but can also modify parameters as the urban green space continuously changes. The formation of a complete evaluation and output improvement process can accelerate the value expansion and implementation of simulation evaluation software based on IoT technology throughout the entire life cycle of the built environment. As a result, our proposed model is more accurate since it can continuously modify its output results and give feedback suggestions as the environmental data changes.

## 2. Model Design and Implementation

**Figure 1:**
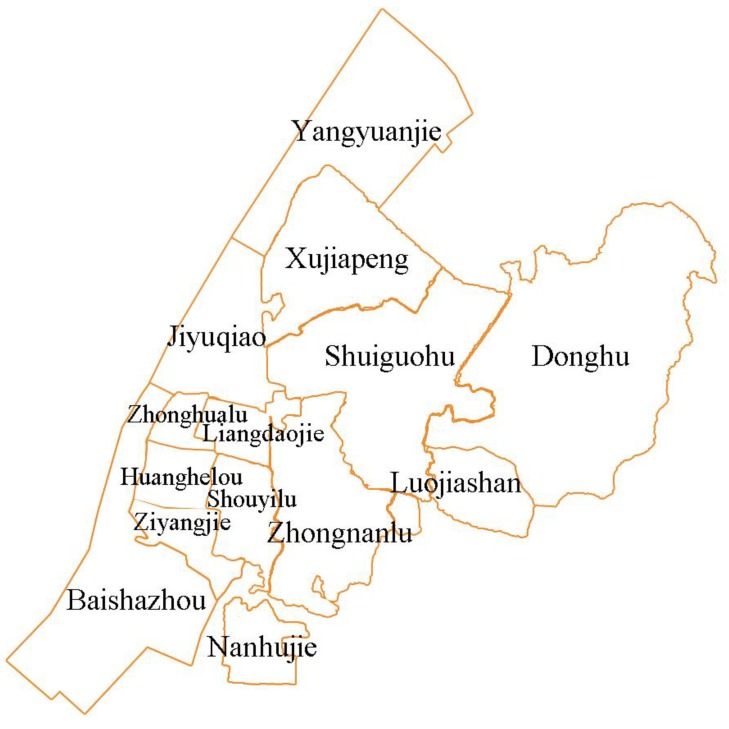
Schematic diagram of the administrative boundary of Wuchang District.

This study selects Wuchang District, Wuhan City, Hubei Province, as the research object. Wuchang District is the seat of the provincial capital of Hubei and the political, cultural, and educational center of Hubei Province. It is located in the southeast of Wuhan, which is one of the central urban areas of Wuhan and one of the jurisdictions with the largest permanent population in Wuhan. The long historical origins and dense urban population of the region make it important and valuable to assess its urban green spaces, thereby promoting realization of sustainable and smart cities. According to the Statistics on Economic and Social Development of Wuchang District, Wuhan City, as of December 2016, there were 14 streets in Wuchang District with a total area of 107.76 square kilometers, including 10.7 square kilometers in the Yangtze River and 3.3 square kilometers in the Shahu water. The East Lake waters cover 32.8 square kilometers, with a total population of 1,135,869 at the end of the year and a population density of 19,727 people/square kilometers. Among them, this study excludes Shidong Street because it has a small land area and a small population and is far from the concentrated distribution of Wuchang District. This study mainly uses 13 streets in Wuchang District except Shidong Street and the Donghu Ecological Tourism Scenic Area as basic units, as shown in Figure 1.

When assessing the perceptions of residents of sustainable and smart cities, the most common method is the ecosystem service (ES) [16], which mainly includes 6 projects [17], including runoff control, air purification, cooling, carbon sequestration, noise reduction, and recreational area establishment. In the field of construction, a two–dimensional indicator of the building density or the proportion of the building land area in a region is used to indicate the flat–land occupation of the building, and the ratio of the total building area to the total area, i.e., the plot ratio, is used to describe the construction intensity. When the building density remains constant, the higher the floor area ratio is, the more building layers there are. For the resident–perceived provider, i.e., the urban green space, at present, there are only indicators such as the green space rate and green coverage, and the lack of three–dimensional measurement indicators such as the plot ratio makes it difficult to rationally determine the green space construction intensity. In this study, the green plot ratio was introduced to evaluate the existing green space construction. Therefore, this study built a multidimensional evaluation index model, which evaluated the comfort level of the urban green space built from the actual experience of residents, and modeled and analyzed it from the perspective of the three–dimensional green volume to provide urban residents with long–term stable and comfortable environments called sustainable and smart cities.

### 2.1. Model Framework

**Figure 2:**
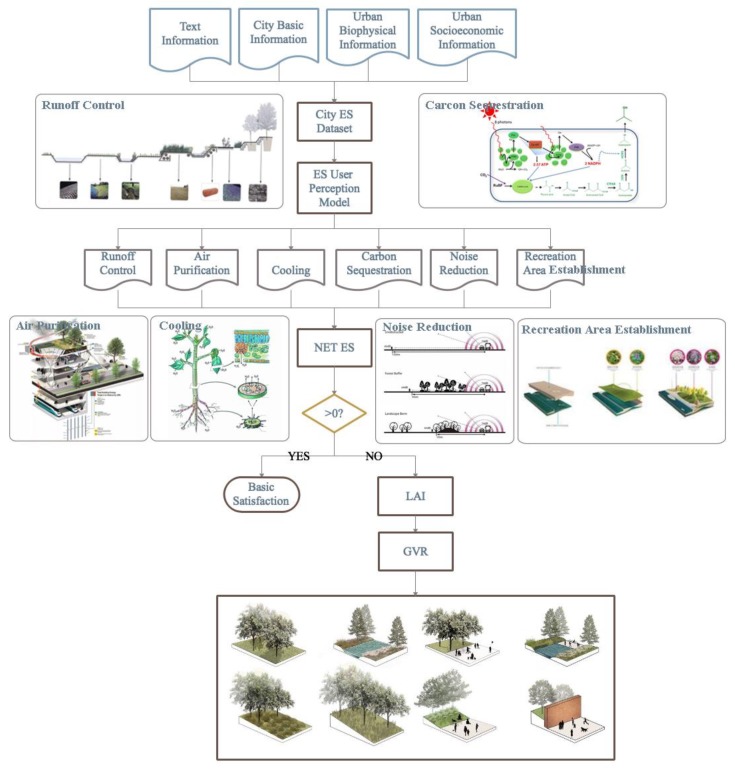
Proposed model architecture diagram.

The model constructed in this study aimed to meet the requirements of sustainable and smart cities. It first required relevant text data, basic city information, urban biophysical information, and urban socioeconomic information to generate urban ES datasets. Second, ES resident perception analysis was performed from the above 6 aspects, targeting different streets, which generated a unique Net ES data set for each street. Finally, urban GVR data were further generated through corresponding street leaf area index (LAI) data, and urban green space improvement suggestions of residents were presented. The workflow is shown in Figure 2, and the following section describes the various subdivision operation steps.

The whole framework can be divided into two stages. The first stage is to collect datasets. The text information, city basic information, urban biophysical information, and urban socioeconomic information were collected by an RE–3DSG sensors. It consisted of ES Resident Perception Sensors and GVR Evaluation Sensors. ES Resident Perception Sensors integrates 6 parts, namely gas sensor, chemical element sensor, sound sensor, humidity sensor, temperature sensor, and recreation sensor. For detail, air purification data were loaded into a gas sensor, carbon sequestration data were loaded into a chemical element sensor, runoff control data were loaded into a humidity sensor, cooling data were loaded into a humidity sensor, noise reduction data were loaded into a sound sensor, and recreational area establishment data were loaded into a recreation sensor (summarized in Table 1). The second stage is to get the feedback of the urban green space. Combined with the structure type of green areas provided by GVR Evaluation Sensors, the model formulated green space adjustment strategies for specific residents (which can also be called urban streets).

### 2.2. ES Resident Perception Sensors

We applied ES Resident Perception as an important sensor, and it was closely correlated to real residents’ feelings on urban green space. In this stage, the ES Resident Perception can be separated into 6 parts: Gas sensor, chemical element sensor, sound sensor, humidity sensor, temperature sensor, and recreation sensor. Then, the properties of the urban biophysical and socioeconomic information, as well as the bilateral relationship with each other, were extracted. Finally, the model was generated based on the obtained information using the ES Resident Perception Sensors.

#### 2.2.1. ES Requirements Sensors

##### Gas Sensor—Air Purification

In this study, PM_10_ was the research object because it causes the most harm to the health of urban residents and can be captured by green spaces. The space requirement for air purification is equivalent to the actual PM_10_ pollution observed. The calculation method is the difference between the PM_10_ concentration of each street and the allowable PM_10_ concentration set by the local government target. If the actual concentration exceeds the allowed PM_10_ concentration, the requirement is the difference between the actual and allowed concentrations. Otherwise, the demand will be zero [17]. The calculation equation is as follows [18]:

(1) $$\{\begin{array}{cc} if\;\rho_{pm,block}\leq PM_{10,permitted}, & PM_{10,block}^{D}=\left(\rho_{pm,block}-PM_{10,permitted}\right)\times H\times A_{block}\\ if\;\rho_{pm,block}>PM_{10,permitted}, & 0 \end{array}$$

where $\mathrm{PM}10_{\mathrm{block}}^{D}$ is the basic air purification requirement, and its unit is kg, $\rho_{\mathrm{pm},\mathrm{block}}$ is the PM_10_ concentration in each street, and the evaluating unit is kg/m^3^; *H* represents the estimated air column, and the troposphere below 200 m is selected as the boundary layer. *A_block_* is the area of each street in m^2^, and $PM_{10,permitted}$ is the maximum permitted PM_10_ concentration.

##### Chemical Sensor—Carbon Sequestration

For the estimation method of carbon dioxide emissions, we refer to the 2006 IPCC Guidelines for National Greenhouse Gas Inventories and obtain the per capita carbon dioxide emissions. The calculation method used in our study for the proportion of carbon converted to carbon dioxide was calculated per g carbon, which is equivalent to 3.667 g carbon dioxide. For the data of the various types of energy consumption in Wuchang District in this study, we refer to the Wuhan Statistical Bureau, Wuhan Statistical Yearbook 2016, published by China Statistics Press (2016). The equation is as follows:

(2) $$CS_{block}^{D}=\frac{E_{i}\times t_{ci}\times \frac{44}{12}}{P}\times P_{block}\times \frac{1000}{3.667}$$

where *E_i_* is the consumption volume of energy type *i*, *t_ci_* is the carbon emission factor for energy type i, $\frac{44}{12}\;$ is the conversion coefficient between carbon and carbon dioxide, *P* is the total population, and *P_block_* is the population in each street.

##### Sound Sensor—Noise Reduction

Recent research shows that noise pollution has become a serious environmental problem in urban areas due to road networks and high population densities, which have caused a certain degree of harm to the daily life and physical and mental health of urban residents. According to the 2017 Wuhan Motor Vehicle Exhaust Pollution Prevention Annual Report released by the Wuhan Municipal Environmental Protection Bureau, the number of motor vehicles in Wuhan in 2017 was 2.878 million, an increase of 10.26% over the previous year. Traffic noise has become the main source of environmental noise. Therefore, this study used traffic–induced noise as the source of the noise reduction demand. Cadna/A is noise simulation software based on the German RLS 90 general calculation model. The calculation principle is based on ISO9613–2: 1996, Calculation Method for Attenuation of Outdoor Sound Propagation, issued by the International Organization for Standardization. It functions the same as the calculation method of sound propagation attenuation and can be popularized in China.

**Figure 3:**
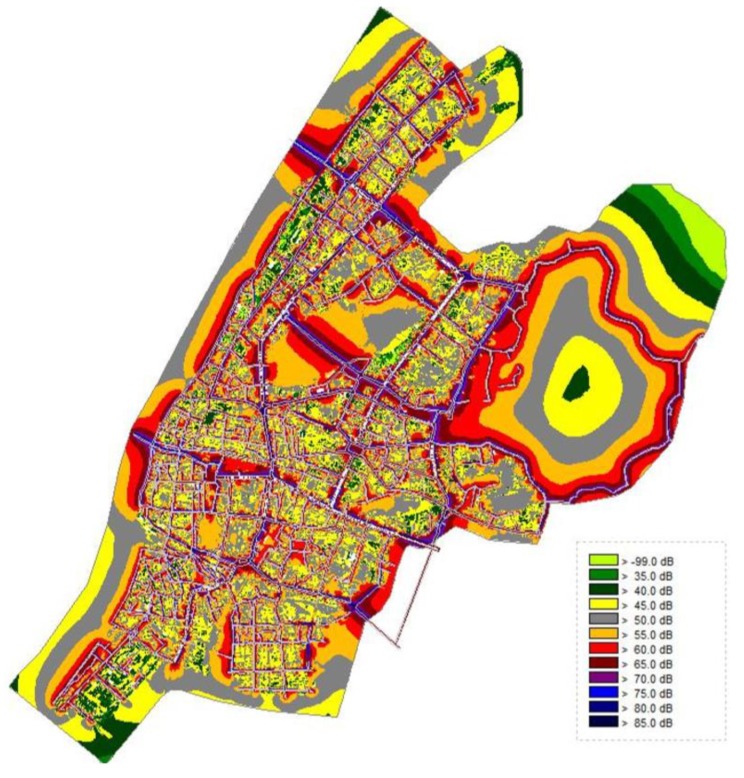
Noise simulation results.

The basic data required for the study included basic traffic flow information, such as vehicle flow, and attribute information of roads and surrounding buildings, such as road names, widths, and building heights. The following conditions are applicable: Horizontal distance of 25 m, smooth asphalt road surface, speed limit of 100 km/h, slope < 5%, sound waves travel freely at an average height of 2.25 m from the road surface. Highway traffic noise prediction in the software can be calculated as follows:

(3) $$L_{m}=37.3+10\times lg\left[m\times \left(1+0.082\times P\right)\right]$$

where *L_m_* is the average sound level and *m* is the average traffic volume per hour in a single lane. When multilane highway calculations are performed, the traffic volume in the two outer lanes is ½ *m*, and *P* is the proportion of heavy vehicles (load capacity > 2.8 tons).

(4) $$L_{m,E}=L_{m}+D_{r}+D_{stro}+D_{stg}$$

where *L_m,E_* is the radiated sound level, *D_r_* is the corrections at different maximum speeds, *D_stro_* is the correction for different road surfaces, and *D_stg_* is the correction for different road slopes. The noise simulation results considering source classification in the sensor [19] are shown in Figure 3.

##### Humidity Sensor—Runoff Control

In this study, runoff control was defined as the combined effect of rainfall interception, infiltration, and water storage. For extreme rainfall events, the ESs provided by green spaces are of great significance, and under moderate rainfall, these services can reduce rainwater treatment and drainage costs [20]. The Ministry of Housing and Urban–Rural Development has issued the Technical Guide for Sponge City Construction–Construction of Low–Impact Development Rainwater Model (Trial) and has adopted the annual runoff control rate as the demand for runoff control in the region. The annual total runoff control rate is defined as the proportion of the total annual controlled (not drained) annual rainfall in urban areas through green space infiltration, evaporation (tap), retention, and storage. The rainfall data source is the 2016 Annual Climate Evaluation Report issued by the Hubei Meteorological Bureau, and the annual runoff control rate data come from the Special Plan for Wuhan Sponge City (2016–2030) issued by Wuhan Planning Bureau.

##### Temperature Sensor—Cooling

The actual cooling demand of urban residents can be measured through the concept of the social environmental risk, which is composed of the social vulnerability [21] and the harm of impact factors. Social vulnerability refers to the number of exposed people and particularly sensitive people in each street, and the number of exposed people corresponds to the statistical population of each street. We consider that elderly individuals older than 60 years are particularly sensitive to heat stress because the temperature regulation ability generally decreases with age, and there is a strong correlation between age and incidence, which in turn increases heat stress sensitivity. This study focuses on summer temperatures, as summer residents have the highest cooling demand. Based on data from 13 county–level stations in Wuchang District of the China Weather Network, the average maximum and minimum temperatures in August 2018 were calculated as follows:

(5) $$HS_{block}^{D}=\left[\frac{P_{block}}{A_{block}}\times \max{\left(\frac{P_{block}}{A_{block}}\right)}^{-1}\times 10\times 0.7+\frac{P_{block}^{60+}}{P_{block}}\times \max{\left(\frac{P_{block}^{60+}}{P_{block}}\right)}^{-1}\times 10\times 0.3\right]\times \left(T_{mean}-T_{min}\right)$$

where *A_block_* is the area of each street, $P_{block}^{60+}$ is the population older than 60 years in each street, *T_mean_* is the average highest temperature in August in the research area, and *T_min_* is the average minimum temperature in August in the research area.

##### Recreation Sensor—Recreation

The most distinct perception of green spaces of urban residents pertains to recreational and entertainment functions. Related research shows that the urban green space becomes more attractive when it provides sufficient recreational and entertainment functions. Wuhan Municipal People’s Government released the Implementation Plan for the Establishment of a National Ecological Garden City in Wuhan on June 16, 2018, stating that the per capita park green area should not be smaller than 10 m^2^/resident. This article uses this value as the standard for the recreational demand and multiplies it by the statistical population of each street to obtain the recreational demand within the scope of this study, which is calculated as follows:

(6) $$RA_{block}^{D}=GV\times P_{block}$$

where *GV* is the per capita green area of parks recommended by the local government (m^2^/resident).

#### 2.2.2. ES Supply Sensors

The main methods for the provision of ESs in this article are as follows: First, the six ESs described above that are closely related to the health and wellbeing of urban residents are quantified. These six services are provided through seven different types of urban green spaces (Table 2). The supply of ES for each green space is calculated by multiplying the area of each element by the supply factor of the corresponding ES type (Table 3) [22]. Second, high–resolution remote sensing images are used to draw supply maps at the city street scale to determine the spatial distribution of supplied ESs.

**Figure 4:**
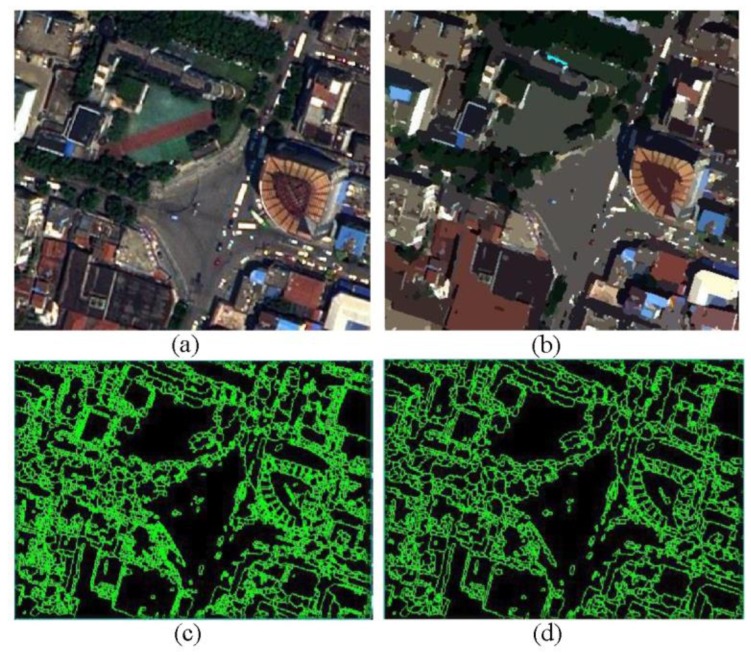
(**a**) the top left picture represents WorldView image before segmentation; (**b**) the top right picture represents WorldView image after segmentation; (**c**) the bottom left picture represents extraction results before image segmentation; (**d**) the bottom right picture represents extraction results after image segmentation.

The data were sourced from the 0.5 m panchromatic and 1.8 m multispectral images provided by the Digitalglobe new–generation commercial imaging WorldView–II satellite. The shooting time was July 29, 2016. On this day, vegetation growth was the best, and the data quality was the highest. As shown in Figure 4, the point cloud generated from ES supply Sensor was very noisy even after the systemic error calibration, which makes most elements extraction methods unsuitable for this situation. We utilized the ENVI–based FX extension module platform for object–oriented classification and extraction of remote sensing images.

Compared with traditional spectral–information–based supervised classification and expert–knowledge–based decision tree classification, this method is widely used in high–resolution remote sensing image extraction due to its higher accuracy in terms of spectrum, texture, and spatial attributes. Object–oriented feature extraction was performed on image data that were preprocessed by image fusion, correction, and enhancement through image segmentation and merging, and the extraction results were adjusted based on field survey results, as shown in Figure 4. In particular, when classifying land in cities, buildings refer to all impervious surfaces, including residential land, commercial land, industrial land, transportation land, etc., while excluding roads.

#### 2.2.3. Net ES

In this study, we assumed that each ES is of equal importance and normalized the value of a single ES from 0 to 10, which can be calculated as follows:

(7) $$\sum NetSupply=\sum_{UES=1}^{6}ES_{\sup ply}\times {\left(\max ES_{\sup ply}\right)}^{-1}\times 10$$

(8) $$\sum NetDemand=\sum_{UES=1}^{6}ES_{demand}\times {\left(\max ES_{demand}\right)}^{-1}\times 10$$

(9) $$NetES=ES_{\sup ply}-ES_{demand}$$

where *NetSupply* is the net supply value, *NetDemand* the net requirement value, NetES is the Net ES value, *ES_supply_* is the supply value of an individual service, and *ES_demand_* is the demand value of an individual service.

### 2.3. GVR Evaluation Sensors

At present, research on the three–dimensional green amount mainly focuses on measuring the leaf area [23]. In the process of measuring the green leaf area, the leaf area in each unit area can be obtained at the same time, called the LAI. Higher LAI values and larger total areas of plant leaves per unit area indicate a richer plant coverage and structure. LAI can be used to evaluate the structure type of green areas, and it becomes an indicator to measure the strength of green land construction, similar to the plot ratio in the field of architectural urban planning, which indicates the construction intensity of an area. We defined LAI as GVR in urban green space assessment, which is the ratio of the total leaf area of the green space to the land area. GVR expresses the intensity of the urban three–dimensional green amount connected with related disciplines such as urban planning and architecture in terms of semantics and regulation [24]. The calculation of GVR is given below.

(10) $$GVR=\frac{\mathrm{LA}}{S}$$

where *LA* is the total leaf area (green amount) in a specific area, and *S* is the total land area. The green plot ratio plays an important role in urban green space evaluation. The green plot ratio can intuitively and accurately discriminate the construction intensity and community structure type of urban green spaces but can also overcome the deficiency of two–dimensional evaluation indicators. The green amount is closely related. After GVR is obtained, the total green amount in a specific area can be calculated accordingly.

In this stage, the GVR Evaluation Sensors operation mode mainly includes 4 tasks. First, selecting the community structure according to its type, e.g., single–layer structure, double–layer structure, and three–layer structure; investigating a certain number of each type of green areas in the research field. Second, investigating the sample areas where the planting time is long, vegetation growth is good, and community structure is stable, and excluding newly planted plants in the past two years. Third, the sample area should be at least 30 × 30 m wide and the community structures should be relatively similar to each other within 30 m, because GPS error factors and edge effects may influence the results. Last, forwarding the relevant feedback suggestions based on the results.

## 3. Performance and Discussion

**Figure 5:**
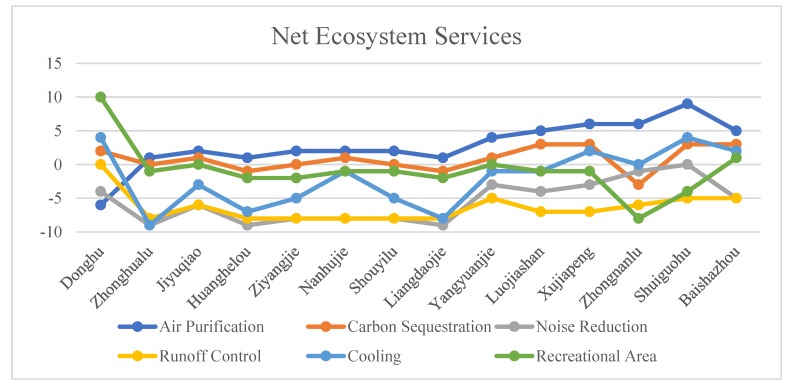
Net ecosystem services results.

**Figure 6:**
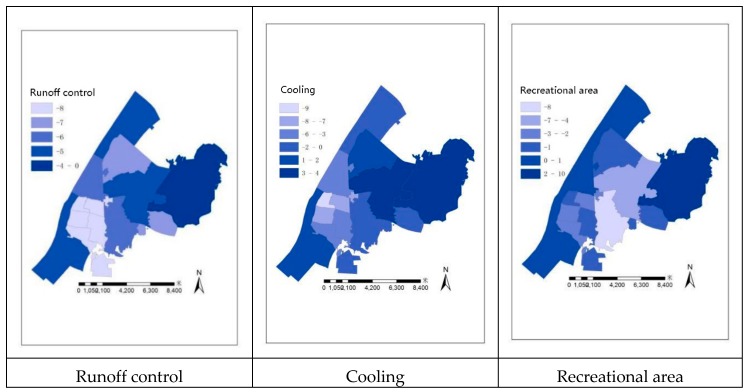

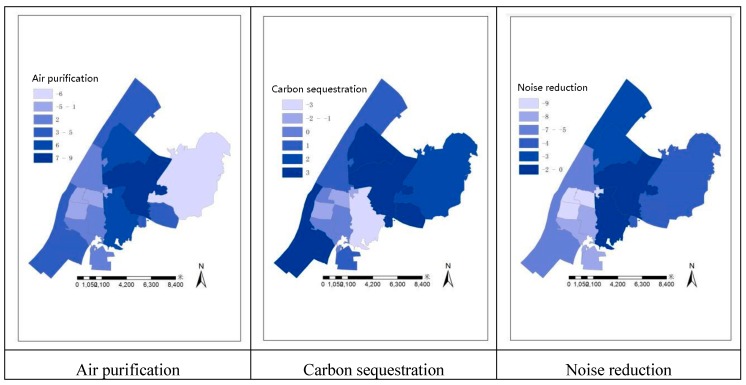
Visualizing the net ecosystem service (ES) results.

The Net ES performance results from implementing our presented model are shown in Figure 5 and Figure 6. The values indicate the usage experience of residents. Negative values represent an insufficient environmental supply, indicating how much residents feel unsatisfied. Positive values indicate that the environmental supply can meet the actual needs of residents and contribute to the construction of sustainable and smart cities. The best comprehensive performance occurred in Shuiguohu Street; the worst comprehensive performance was observed in Liangdao Street, Zhonghua Road Street, Huanghelou Street, Ziyang Street, and Shuyi Road Street, which contained a large number of shantytowns and old residential buildings, and their own green spaces were comparatively smaller. In addition, Yangyuan Street, Xujiapeng Street, Baishazhou Street, and Luojiashan Street performed well in air purification and carbon sequestration, and their other services performed at the medium level. Jiyuqiao Street and Nanhu Street performed poorly in noise reduction, and the performance of this service was at a medium level. The performance of recreation and entertainment and carbon sequestration were the worst in Zhongnan Road Street, while the other services were at the medium level. The East Lake Ecological Tourism Scenic Area, with water bodies as the main GI type, performed the best in runoff control and recreation.

**Figure 7:**
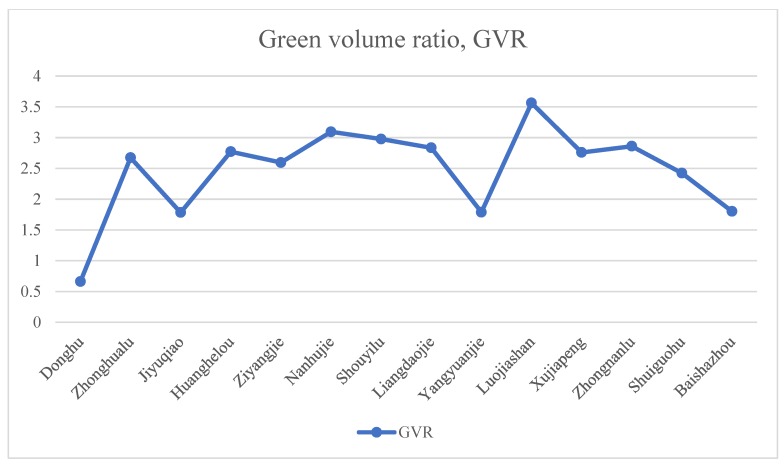
The green volume ratio (GVR) results in 14 areas.

**Figure 8:**
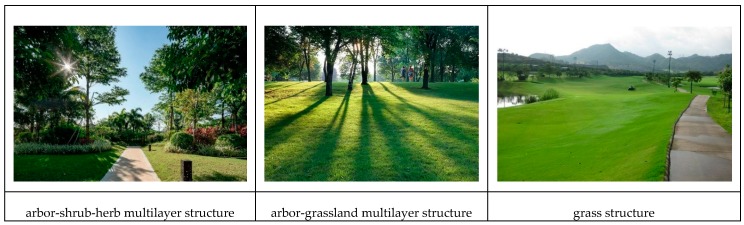
Three types of urban green space.

The GVR results obtained after running the model are shown in Figure 7. GVR reflects the potential level of the urban green space to provide residents with ESs. A higher GVR value indicates that the plants in the green space have a higher density and richer façade level. Generally, the order of GVR is arbor shrub grass multilayer jungle > arbor grass sparse forest grass > single–layer grass (Figure 8). Lujiashan Street had the highest GVR value (3.56), indicating that it had the highest potential for providing ESs. In the future, urban development can focus on dedicating related resources to create a people–oriented park city. In addition, Nanhu Street (3.09), Zhongnan Road Street (2.86), and other streets also had a high potential. It is important to note that the GVR value of Shouyi Road Street, whose value was 2.97, ranked third among the 13 streets in Wuchang District, but its overall ES performance was poor. For such cities, streets with less development space and higher levels of urban development can be transformed in the most suitable way. Each idle small space can be transformed into pocket parks of different sizes to facilitate urban residents. Residents do not have to walk very far to reach these spaces and can spend some time enjoying these parks. In addition, the East Lake Scenic Area had the lowest GVR value, only 0.66, which is related to the fact that more than 90% of its land was composed of water bodies; therefore, the GVR measurement result in most areas was 0. For such scenic spots, the green lung function of the urban green space to maintain water and soil and conserve water resources was fully utilized, while large–scale development was reduced.

The key to determining the accuracy of this study is whether the urban green space classification system is correct, because the basic data of the model operation were derived from the urban green space classification system. As a result, we applied confusion matrix for comparing the difference between classification results and ground truth information, and for displaying the accuracy of the classification results to verify the effectiveness of the model results. A confusion matrix is a visual classification effect diagram in the field of pattern recognition, and it depicts the relationship between the true attributes of sample data and the type of recognition results. A confusion matrix can be applied to evaluate the performance of classifiers, e.g., assuming that for the classification task of N types of patterns, the recognition data set D includes T0 samples, and each type of pattern contains Ti data (i = 1, 2, 3...N). A recognition algorithm was used to construct the classifier C. CMij indicates the percentage that ith pattern in sample data can be determined to the jth pattern by the classifier C among total number of samples of the ith pattern obtained as an N × N dimensional confusion matrix, shown in Equation (11).

(11) $$CM\left(C,D\right)=\left\{\begin{array}{ccc} \begin{array}{cc} \begin{array}{c} cn_{11}\\ cn_{21}\\ \vdots \end{array} & \begin{array}{c} \cdots \\ \ldots \\ \ddots \end{array} \end{array} & \begin{array}{c} cn_{1i}\\ cn_{2i}\\ \vdots \end{array} & \begin{array}{cc} \begin{array}{c} \ldots \\ \ldots \\ \ddots \end{array} & \begin{array}{c} cn_{1N}\\ cn_{2N}\\ \vdots \end{array} \end{array}\\ \begin{array}{cc} cn_{i1} & \ldots \end{array} & cn_{ii} & \begin{array}{cc} \ldots & cn_{iN} \end{array}\\ \begin{array}{cc} \begin{array}{c} \vdots \\ cn_{N1} \end{array} & \begin{array}{c} \ddots \\ \cdots \end{array} \end{array} & \begin{array}{c} \vdots \\ cn_{Ni} \end{array} & \begin{array}{cc} \begin{array}{c} \ddots \\ \ldots \end{array} & \begin{array}{c} \vdots \\ cn_{NN} \end{array} \end{array} \end{array}\right\}$$

where diagonal elements represent the percentage of each pattern that can be correctly recognized by classifier C, while non–diagonal elements represent the percentage of false classifications. Through the confusion matrix, the correct recognition rate and incorrect recognition rate of the required verification content can be obtained. Equation (12) demonstrates correct recognition rate of each mode.

(12) $$R_{i}=cn_{ii},\;\;i=1,\ldots ,\;N$$

Error recognition rate of each mode can be computed as Equation (13).

(13) $$W_{i}=\sum_{i=0,j\neq 0}^{N}cm_{ij}=1-cm_{ij}=1-R$$

We calculated the confusion matrix for seven types of data in ES supply Sensors. The results were arbor tree correct rate was 81.3%, woodland correct rate was 82.6%, shrub correct rate was 77.1%, herb correct rate was 89.6%, garden correct rate was 85.3%, the accuracy rate of water body was 91.0%, and the accuracy rate of others was 84.7%. In general, the classification results were distributed in the range of 80% to 90%. Compared with conventional supervised classification and decision tree classification based on expert knowledge, the accuracy was higher and the classification of urban land was more accurate. The highest accuracy of the water body (91.0%) was due to the large difference between the spectral attributes of other land types and could therefore be more easily identified. The arbor tree and woodland had the same spectral and object attributes except spatial attributes, so they were easily misclassified with a low accuracy rate. Because the number of shrubs was small and usually did not exist in combination with arbor tree, woodland, and herb, the recognition accuracy rate was the lowest. We are looking forward to improving its recognition accuracy rate in the subsequent research.

## 4. Conclusions

In this paper, we propose a multidimensional assessment model by using RE–3DSG sensors on Net ES and GVR for sustainable and smart cities. Specifically, it modifies the parameterization and visualization functions of the traditional technology and utilizes relevant data related to the actual experience of residents to conduct 3D evaluation of the urban green space structure of a city, with the final results visualized. Using this model can reduce the gap between virtual simulation and actual conditions, obtain more accurate first–hand urban green space data, and thus support sustainable and smart city construction. In terms of the validity of the Net ES results, on the one hand, users (usually decision makers in government departments) can verify the accuracy of the aforementioned results through the leaf area (LA), and on the other hand, they can also intuitively evaluate whether the current urban green space environment meets the needs of residents through field research. When Net ES is positive. The model considers that the urban green space has basically satisfied the needs of residents, and when Net ES is negative, it considers that the urban green space cannot meet the basic requirements of residents, and it will further calculate the GVR value of the area and propose corresponding improvement measures according to the actual situation. In terms of supporting sustainable and smart city construction, there is no clear linear relationship between Net ES and GVR. For urban areas that have been relatively well developed, urban green spaces can be expanded from a three–dimensional perspective when the urban land area is limited. When the GVR value is between 2.5 and 3.5, the potential of the urban green space is very large, and when the GVR value is lower than 1, it is difficult to improve the urban green space from a three–dimensional perspective, and other research aspects should be considered.

This study proposes a multidimensional assessment model by using RE–3DSG sensors on Net ES and GVR. It has the advantages of a simple operation and easy data acquisition. At the same time, it bridges the gap in traditional environmental evaluation models that do not consider the actual experience of residents and three–dimensional construction. It incorporates various types of city data into the calculation process and generates final feedback suggestions to provide users (usually decision makers in government departments) with a decision–making reference, to improve the decision–making efficiency and to meet the actual space needs of residents. In the future, the model will cover increasing amounts of extensive data to assist the construction of sustainable and smart city environments.

## Figures and Tables

**Table 1:** Indicator description and data source.

Function	Service Type	Measure Demand Standard	Data Sources
air purification	adjustment	Spatialization of the annual average PM_10_ concentration	Monitoring data of the Wuhan Ecological Environment Bureau
cooling	adjustment	Combining the overall vulnerability of the population density, the proportion of elderly individuals and the highest cooling trend of the region	Data from the Wuhan Regional Climate Center, and demographic data from the Wuchang District Bureau of Statistics (2016)
carbon sequestration	adjustment	Spatialization of the per capita annual CO_2_ emissions (kg)	Wuhan Statistical Yearbook of Various Energy Consumption (2016), Wuchang District Bureau of Statistics Population Statistics
noise reduction	adjustment	Noise simulation weighted average (dB)	Wuhan Urban Planning Bureau Road Network Traffic Data (2016), road vector data, Baidu panoramas
runoff control	adjustment and supply	Annual runoff control rate	Technical Guide for Sponge City Construction–Low Impact Development of Rainwater Model Construction (Trial), 2016 Annual Climate Evaluation Report, Special Plan for Wuhan Sponge City (2016–2030)
recreational area establishment	culture	Spatialization of the per capita park green space proposed by the government to create a national ecological garden city (m^2^)	Demographic data of the Wuchang District Bureau of Statistics (2016), Implementation Plan for Creating a National Ecological Garden City in Wuhan

**Table 2:** Green infrastructure (GI) category description.

GI Category	Definition	Data Source	Data Type
arbor tree	A single independent tree, especially a sidewalk tree, usually surrounded by paved ground	Remote sensing image	Points
woodland	Density of trees and urban forest	Remote sensing image	Flat
shrub	Shrub or hedge	Remote sensing image and field research	Flat
herb	Vegetation types mainly include nonwoody plants such as herbs	Remote sensing image	Flat
garden	Sealed surfaces with vegetation and water around, such as ecological parking lots, permeable paving squares.	Remote sensing image and field research	Flat
water body	Open waters and flowing water bodies such as rivers, lakes and ponds	Remote sensing image	Flat
others	Sports fields, playgrounds, golf courses, urban unused land, etc.	Remote sensing image and field research	Flat

**Table 3:** Supply type and coefficient table.

GI Type	Air Purification	Carbon Sequestration	Noise Reduction	Runoff Control	Cooling	Recreation
arbor tree	3.97	10.64	N/A	8.4	1	2.15
woodland	2.69	15.62	1.125	8.7	1	2.9
shrub	2.05	7.79	2	7.3	1	2.55
herb	0.9	0.17	0.375	8	0.5	2.55
garden	0.82	1.07	N/A	6	0.5	N/A
water body	N/A	N/A	N/A	10	N/A	2.2
others	0.82	1.07	0.375	6	0.5	2.35
